# Yi Shen Juan Bi Pill Regulates the Bone Immune Microenvironment *via* the JAK2/STAT3 Signaling Pathway *in Vitro*

**DOI:** 10.3389/fphar.2021.746786

**Published:** 2021-12-14

**Authors:** Ya Xia, Danping Fan, Xiaoya Li, Xiangchen Lu, Qinbin Ye, Xiaoyu Xi, Qiong Wang, Hongyan Zhao, Cheng Xiao

**Affiliations:** ^1^ School of Traditional Chinese Medicine, Beijing University of Chinese Medicine, Beijing, China; ^2^ Department of Emergency, China-Japan Friendship Hospital, Beijing, China; ^3^ Graduate School of Peking Union Medical College, Chinese Academy of Medical Sciences/Peking Union Medical College, Beijing, China; ^4^ Institute of Clinical Medicine, China-Japan Friendship Hospital, Beijing, China; ^5^ The Institute of Medicinal Plant Development, Chinese Academy of Medical Sciences/Peking Union Medical College, Beijing, China; ^6^ Pinggu Hospital, Beijing Traditional Chinese Medicine Hospital, Beijing, China; ^7^ Beijing Key Laboratory of Research of Chinese Medicine on Prevention and Treatment for Major Diseases, Experimental Research Center, China Academy of Chinese Medical Sciences, Beijing, China

**Keywords:** rheumatoid arthritis, Yi Shen Juan Bi Pill, osteoclast, regulatory T cell, JAK2/STAT3 signaling pathway

## Abstract

Rheumatoid arthritis (RA) is characterized by an impaired articular bone immune microenvironment, which is associated with regulatory T cells (Tregs) hypofunction and osteoclasts (OCs) hyperfunction and leads to articular bone erosion and systemic bone loss. Studies have shown that Tregs slow bone loss in RA by regulating the bone resorption function of OCs and the JAK/STAT signaling pathway can regulate the immunosuppressive function of Tregs and reduce the bone erosion function of OCs. Yi Shen Juan Bi Pill (YSJB) is a classic Chinese herbal compound for the treatment of RA. However, whether YSJB regulates bone immune microenvironment homeostasis through JAK/STAT signaling pathway remains unclear. Based on *in vitro* OC single culture, Treg single culture and OC-Treg coculture systems, treatments were performed using drug-containing serum, AG490 and JAK2 siRNA to explore whether YSJB-containing serum regulates the homeostasis of the bone immune microenvironment through the JAK/STAT signaling pathway. *In vitro*, YSJB treatment decreased the number of TRAP^+^ cells and the areas of bone resorption and inhibited the expression of RANK, NFATc1, c-fos, JAK2, and STAT3 in both the OC single culture system and the OC-Treg coculture system. Tregs further reduced the number of TRAP^+^ cells and the areas of bone resorption in the coculture system. YSJB promoted the secretion of IL-10 while inhibiting the expression of JAK2 and STAT3 in Tregs. Moreover, inhibiting the expression of JAK2 with the JAK2 inhibitor AG490 and JAK2 siRNA improved the immunosuppressive functions of Treg, inhibited OC differentiation and bone resorption. Our study demonstrates that YSJB can regulate OC-mediated bone resorption and Treg-mediated bone immunity through the JAK2/STAT3 signaling pathway. This study provides a new strategy for regulating the bone immune microenvironment in RA with traditional Chinese medicine.

## Introduction

Rheumatoid arthritis (RA) is a chronic autoimmune inflammatory disease that is characterized by irreversible cartilage injury and secondary bone erosion. Most RA patients develop into disability following bone destruction and bone loss (Smolen et al., 2016; Rotta et al., 2020). Bone damage and bone loss are closely associated with abnormal bone resorption mediated by osteoclasts (OCs) (Zhao et al., 2017). In addition, overactivation of the immune system disrupts bone homeostasis. As negative immunoregulatory cells, regulatory T cells (Tregs) are particularly important for regulating bone balance in RA. Our previous study also showed that Tregs inhibited the differentiation and bone resorption of OCs by secreting interleukin (IL)-10 and transforming growth factor (TGF)-β, which regulated bone immune microenvironment homeostasis (Xu et al., 2016).

The Janus kinase/signal transduction and activator of transcription (JAK/STAT) signaling pathway, which acts as a downstream element of more than 50 cytokines, not only regulates embryonic formation and the immune response but also affects biological processes such as cell proliferation, differentiation, and apoptosis, including those of OCs and Tregs (Villarino et al., 2017). As a specific JAK2 kinase inhibitor, AG490 blocks RANKL-mediated OC differentiation by inhibiting the expression of NFATc1 *via* the STAT3 pathway (Li et al., 2013). Moreover, AG490 inhibits OC differentiation by downregulating the number of CD4^+^IL^-^17A^+^ T helper 17 (Th17) cells and upregulating the number of CD4^+^CD25^+^forkhead box P3 (Foxp3)^+^ Tregs (Park et al., 2014), as well as several molecules that favor Treg functions, such as Foxp3 and programmed cell death protein 1 (ICOS) (Park et al., 2014; Chen et al., 2018; Li and Xiong, 2020).

In traditional Chinese medicine, kidney deficiency syndrome is the most common syndrome of RA. Yi Shen Juan Bi Pill (YSJB), a classic herbal compound in traditional Chinese medicine, has been widely used to treat RA with kidney deficiency patterns in clinical settings in China. We have previously shown that YSJB significantly alleviated bone damage in collagen-induced arthritis (CIA) rats with kidney deficiency and inhibited local joint inflammation and OC differentiation in CIA rats (Zhao et al., 2012). Interestingly, YSJB also regulates the balance of T cell phenotypes (Zhao et al., 2018). In addition, YSJB regulates the levels of IL-6, IL-17A, IL-10, TGF-β1, Th17, Tregs and OCs in CIA rats. These factors and cells are important regulatory factors and effector cells in the JAK2/STAT3 pathway and mediate bone resorption. However, it has not been reported whether the mechanism by which YSJB can treat RA is associated with the JAK2/STAT3 signaling pathway. The present study used CIA rats with castration and obtained YSJB-containing serum. By blocking the JAK2/STAT3 signaling pathway, the mechanism by which YSJB regulates the JAK2/STAT3 signaling pathway to alleviate bone resorption was further explored based on an OC-Treg coculture system. This study provides a new experimental basis for the treatment of RA with YSJB.

## Methods

### Animals

Female Sprague-Dawley (SD) rats (180–200 g) and male C57BL/6 mice (6 weeks old) were purchased from the National Institutes for Food and Drug Control [animal license number: SCXK (Beijing) 2014-0013]. All animals were housed in the Experimental Animal Center of the Institute of Clinical Medical Sciences, China-Japan Friendship Hospital (Experimental Animal Center license number: SCXK (Beijing) 2016-0043). All experiments received ethic approval by the Institute of Clinical Medical Sciences, China-Japan Friendship Hospital, Beijing, China and were conducted within ethical limits. The animals were maintained in a specific pathogen-free environment with a temperature of 23°C (±2°C) and were subjected to a 12 h light/dark cycle.

### Preparation of Drug-Containing Sera

The 40 female rats were randomly divided into two groups according to weight. Then, 32 of the rats were anesthetized, and their ovaries were removed according to standard surgical procedures. The other 8 rats were treated as the control group, in which fat tissue near the ovaries on both sides were removed. Twenty-eight days after castration, the 32 castrated rats were immunized intradermally at the tail root with 100 µl of a fully emulsified mixture of bovine type II collagen (Chondrex, Redmond, United States) and isopycnic incomplete Freund’s adjuvant (IFA; Chondrex) and received booster injections in the same way on day 7 after the initial immunization. Meanwhile, control group rats underwent the same method of injection with an equal volume of saline water.

On the 14th day after immunization, rats that successfully developed arthritis were divided into three groups as follows: 1) castrated CIA, 2) castrated CIA plus methotrexate (MTX; Shanghai Sine Pharmaceutical, Shanghai, China) treatment, and 3) castrated CIA plus YSJB (Jiangsu Zhengda Qingjiang Pharmaceutical, Jiangsu, China) treatment. The rats in the MTX group and YSJB group were treated with MTX (1.02 mg/kg body weight) and YSJB (3.87 g/kg body weight), respectively, by gavage twice a day. Meanwhile, rats in the control group and CIA group received an equal volume of distilled water (10 ml/kg). Rats in each group were given the treatments for 3 days. One hour after the last dose, all rats were anesthetized. Blood samples were collected from the abdominal aorta and centrifuged to obtain serum (quality control data are shown in Supplementary Material S1 and Supplementary Material S2).

### OC Isolation and Culture

OC progenitor cells (OPCs) were isolated and cultured as previously reported (Xu et al., 2016). The mice were sacrificed by cervical dislocation. Femurs and tibias were isolated, and the ends were trimmed. The bone marrow cavity was rinsed with alpha-modified Eagle’s medium (α-MEM; HyClone, Logan, United States), and bone marrow cells (BMCs) were collected by centrifuging the medium containing bone marrow. Subsequently, cells were cultured in α-MEM supplemented with 10% fetal bovine serum (FBS; PAN Biotech GmbH, Aidenbach, Germany), 1% penicillin-streptomycin (PS; Gibco BRL, Grand Island, NY, United States) and recombinant murine M-CSF (20 ng/ml; PeproTech, Rocky Hill, United States) after red blood cell (RBC) lysis buffer (Solarbio, Beijing, China) was used to deplete RBCs. Twenty-four hours later, nonadherent cells were collected and cultured in tissue culture plates at an appropriate density in α-MEM containing 10% FBS and M-CSF (20 ng/ml). Three days later, adherent cells were considered OPCs. To induce OPCs to differentiate into OCs, the cells were cultured for an additional 5 days in fresh differentiation α-MEM containing 10% FBS, 1% PS, M-CSF (20 ng/ml), and RANKL (50 ng/ml; PeproTech). Complete fresh differentiation medium was used every couple of days, and the concentration of each factor remained constant.

### CD4^+^CD25^+^ Treg Purification and Culture

Suspensions of splenic cells were obtained by passing spleen tissue isolated from C57BL/6 mice through a 200-mesh metal mesh grid in RPMI-1640 (Gibco) medium, followed by filtration with a 70 mm cell strainer (BD Biosciences, San Jose, United States). Spleen cells were sorted using an EasySep™ Mouse CD4 Positive T Cell Pre-Enrichment Kit (STEMCELL Technologies, Vancouver, Canada) to obtain CD4^+^ T cells, and then negative selection was used to obtain CD4^+^CD25^−^ Tregs using an EasySep^®^ Mouse CD25 Positive Selection Kit (STEMCELL). CD4^+^CD25^−^ Tregs were resuspended in differentiation RPMI-1640 medium containing 10% FBS, 1% PS, recombinant murine IL-2 (1,000 U/mL; PeproTech), TGF-β1 (10 ng/ml; PeproTech) and anti-CD28 (2 μg/ml; BD Biosciences) and cultured in a round-bottom 96-well plate with anti-murine CD3 (10 μg/ml; BD Biosciences) at a density of 5 × 10^5^ cells/well (200 μl per well). Half of the differentiation medium was refreshed every couple of days and the concentration of each factor remained constant. CD4^+^CD25^+^ Tregs were successfully positively selected with an EasySep^®^ Mouse CD25 Positive Selection Kit after 7 days and were used for subsequent experiments. We explored the method during the early stage. Flow cytometry was used to identify the phenotype of Tregs (Xu et al., 2016).

### Coculture of OCs With CD4^+^CD25^+^ Tregs

Nonadherent BMCs were seeded in 24-well culture plates at a density of 5 × 10^5^ cells/well. Three days later, Tregs (2 × 10^4^) were added to each well. Cells were then maintained in differentiation α-MEM with a 1:25 ratio of Tregs: OPCs. Every 2 days, half of the medium was replaced with fresh differentiation α-MEM. The bottom was not touched when changing medium.

### Groups and the Addition of Drug-Containing Serum

Each culture system was divided into four groups: DMSO, AG490, MOCK and siRNA. DMSO was used as a control for AG490, and MOCK was used as a control for siRNA. Each group was further divided into four subgroups as follows: 1) control, 2) CIA, 3) CIA+MTX, and 4) CIA+YSJB. OPCs and Tregs were cultured in the absence or presence of AG490 (MedChemExpress, New Jersey, United States) and siRNA (SyngenTech, Beijing, China). Twenty-four hours later, 10% drug-containing serum that had been previously obtained from rats was added to the differentiation medium.

### The Cytotoxicity of AG490 Against OCs

The effect of AG490 on cell viability was measured using a CCK-8 assay. OPCs were cultured in 96-well plates at a density of 4 × 10^4^ cells/well in differentiation α-MEM (20 ng/mL M-CSF, 50 ng/ml RANKL, 10% FBS, 1% PS) with AG490 (0, 2.5, 5, 10, 20, 50 μM). The concentration of each factor was kept constant when adding fresh medium. Five days later, fresh α-MEM containing 10% CCK-8 was used to replace the differentiation α-MEM. The absorbance was measured at a wavelength of 450 nm.

### siRNA Interference

siRNA was purchased from SyngenTech. All sequences are shown in Supplementary Material S3. Cells were transfected with siRNA using a Lipofectamine^®^ 3,000 transfection kit (Thermo Fisher Scientific, Waltham, United States) according to the manufacturer’s protocol. qRT-PCR was used to confirm the transfection efficiency.

### Tartrate-Resistant Acid Phosphatase Staining

Nonadherent BMCs were seeded in 96-well culture plates at a density of 1 × 10^5^ cells/well (200 μl per well). After the OPCs were cultured for 5 days, TRAP staining was performed according to the instructions of the TRAP staining kit (Sigma-Aldrich, Louis, United States) to assess osteoclastogenesis. OC formation was scored by the number of TRAP^+^ cells that contained 3 or more nuclei. OCs in the whole well were counted.

### Bone Resorption Pit Formation Assay

Bone slices (Immunodiagnostic Systems Limited, Boldon, United Kingdom) were placed in 96-well culture plates containing nonadherent BMCs at a density of 4×10^5^ cells/well (200 μL per well), incubated for 3 days and cultured with OPCs for 10 days. Then, the bone slices were fixed in 2.5% glutaraldehyde for 5 min and washed in 0.25 mol/L ammonium hydroxide for 10 min to remove the cells from the bone slices. The bone slices were sequentially dehydrated with 40, 75, 95, and 100% ethanol. After drying naturally, the bone slices were dyed with 1% toluidine blue (Sigma-Aldrich) at room temperature for 5 min. Using a Leica Qwin image analysis system (Leica Microsystem, Germany), the areas of the bone resorption pits were analyzed.

### Enzyme-Linked Immunosorbent Assay

Culture supernatant was collected on day 5. According to the instructions of the ELISA kit (Diaclone, France), the levels of IL-10 and TGF-β1 were measured.

### Quantitative Real-Time PCR

Total RNA was isolated using TRIzol reagent (Invitrogen, Carlsbad, CA, United States). A PrimeScript RT reagent kit (TaKaRa, Tokyo, Japan) was used to reverse transcribe RNA into cDNA. The levels of mRNA were analyzed using SYBR Premix Ex Taq (TliRNaseH Plus; TaKaRa) in a Quant Studio 5 real-time PCR instrument (Thermo Fisher Scientific). The thermocycling conditions were as follows: incubation at 95°C for 30 s, followed by 40 cycles of 95°C for 5 s, 60°C for 34 s, then dissociation stage. The results were normalized to β-actin and calculated using the 2^−ΔΔCt^ method. Primer sequences are shown in Supplementary Material S4.

### Western Blot

Proteins of OCs and Tregs were harvested using RIPA Lysis Buffer (Beyotime Biotechnology, Shanghai, China) that was supplied with protease and phosphatase inhibitor cocktails (Beijing ComWin Biotech Co., Ltd. Beijing, China). The protein concentration was measured using bicinchoninic acid (BCA; Solarbio) protein assay. Then, the proteins were mixed with loading buffer and denatured in metal bath (100°C, 5 min). Following, the target proteins were divided *via* sodium dodecyl sulfate–polyacrylamide gel electrophoresis with constant voltage of 100 V and transferred onto a polyvinylidene fluoride membrane (Millipore, Billerica, United States) with constant current of 300 mA in 1 h. In order to eliminate nonspecific protein binding, membranes were blocked at room temperature for 2 h with 5% skim milk (BD Biosciences) in 1 × TBST (Beyotime). And then, membranes were incubated overnight at 4°C with primary antibodies followed: phosphorylated (p-) JAK2 (1:1,000), p-STAT3 (1:2000), JAK2 (1:1,000), STAT3 (1:1,000) (Cell Signaling Technology, MA, United States). Appropriate secondary antibodies were incubated for 2 h and protein bands were detected using eECL western blot kit (Beijing ComWin Biotech Co., Ltd.). Band densities were quantified using Image Lab 3.0 software (Bio-Rad Laboratories, CA, United States).

### Statistical Analysis

Statistical analysis was carried out using SPSS 20.0 software. One-way analysis of variance (ANOVA) and Dunnett’s T3 test were used to determine significant differences. Nonparametric tests were used if the data were abnormally distributed. All data are expressed as the mean ± SEM. The significance level was set at *p* < 0.05.

## Results

### Cytotoxicity of AG490 on OPCs

**FIGURE 1:**
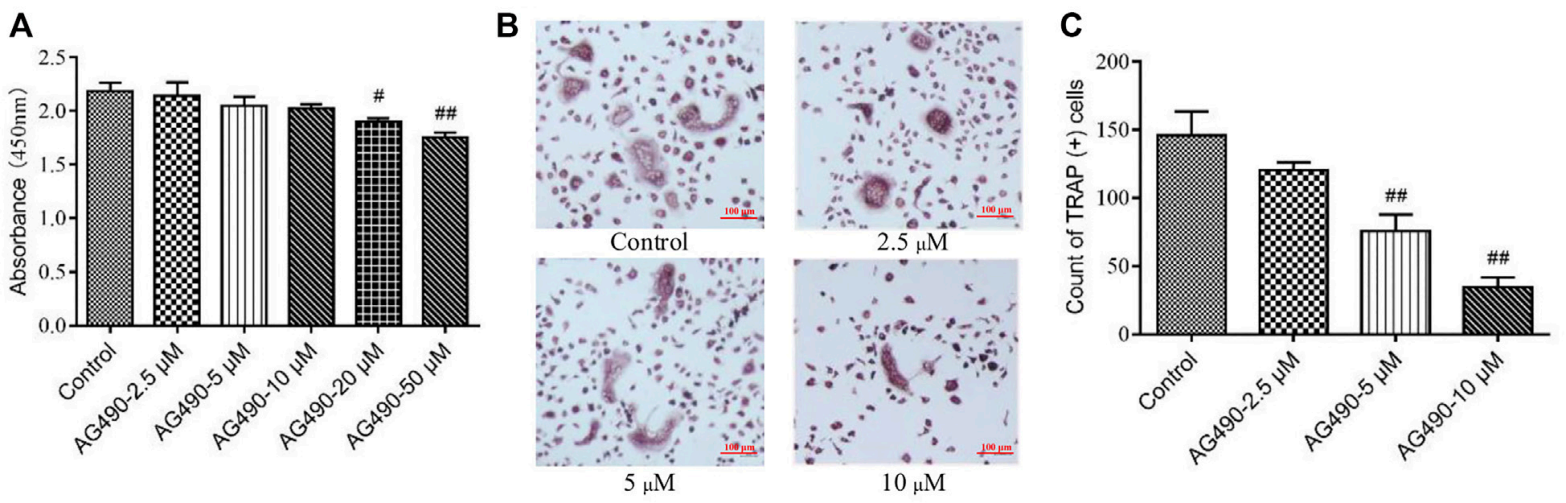
Effect of AG490 on cells viability and osteoclastogenesis in the OC single culture system. **(A)** Cells (OPCs and OCs) viability was assessed by CCK-8 assays after treatment with AG490 (0, 2.5, 5, 10, 20, or 50 μM). **(B,C)** TRAP staining in OCs after treatment with AG490 (0, 2.5, 5 or 10 μM). Scale bars = 100 µm. The data are shown as the mean ± SEM (n ≥ 2). ^#^ *p* < 0.05, ^##^ *p* < 0.01 compared with the control group.

As we all know, OPCs can differentiate into OCs under the stimulation of M-CSF and RANKL (Xu et al., 2016). That’s how we get OCs in this study. Since a paucity of evidence has been found on the toxicity of AG490 in OPCs, we first determined the dose with CCK-8 assays and TRAP staining. OPCs might have differentiated into OCs during CCK-8 assay, so the experiment reflected the effect of AG490 on the viability of overall cells, including OPCs and OCs. The results showed that the absorbance decreased with increasing AG490 concentrations. AG490 had almost no effect on the viability of cells at doses ranging from 2.5 to 10 μM (Figure 1A), which indicated that AG490 was not cytotoxic to cells (OPCs and OCs) at concentrations below 10 μM. Moreover, when the concentration of AG490 was greater than 5 μM, the number of TRAP^+^ cells decreased significantly (Figures 1B,C). The TRAP staining revealed a situation how many OPCs differentiated into OCs. It suggested that AG490 could inhibit the differentiation of OPCs into OCs at concentrations greater than 5 μM. Based on these results, 10 μM AG490 was used for subsequent experiments.

### YSJB Blocked the JAK2/STAT3 Signaling Pathway in Tregs and OCs

**FIGURE 2:**
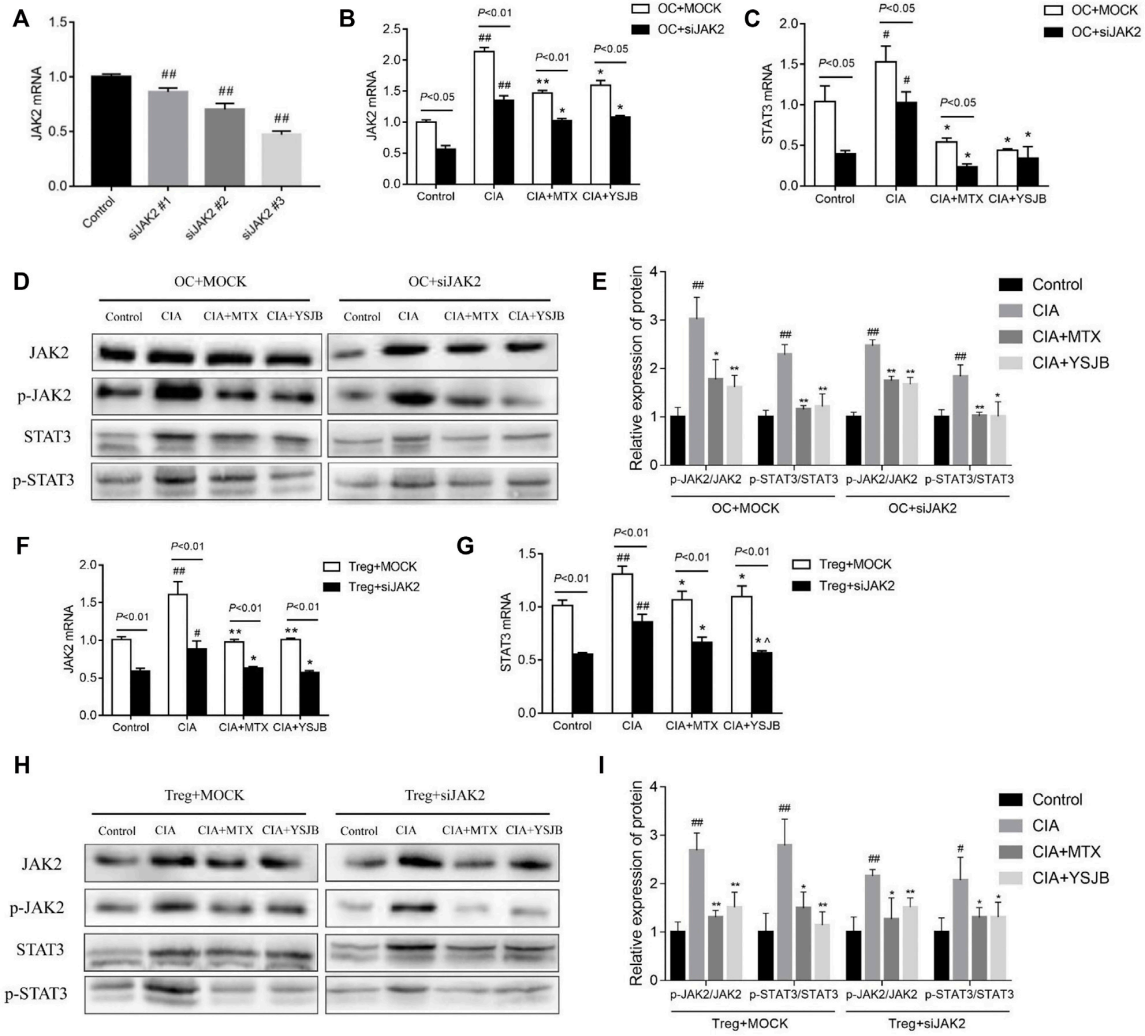
Effect of YSJB and JAK2 siRNA on the JAK2/STAT3 signaling pathway. **(A)** Confirmation of JAK2 knockdown in OCs. **(B)** The relative mRNA levels of JAK2 in OCs. **(C)** The relative mRNA levels of STAT3 in OCs. **(D)** Representative bands of JAK2, p-JAK2, STAT3, and p-STAT3 protein detected by western blot assay in OCs. **(E)** Protein expression levels of p-JAK2/JAK2 and p-STAT3/STAT3 in OCs. **(F)** The relative mRNA levels of JAK2 in Tregs. **(G)** The relative mRNA levels of STAT3 in Tregs. **(H)** Representative bands of JAK2, p-JAK2, STAT3, and p-STAT3 protein detected by western blot assay in Tregs. **(I)** Protein expression levels of p-JAK2/JAK2 and p-STAT3/STAT3 in Tregs. Cells were treated with drug-containing serum and JAK2 siRNA. ^#^ *p* < 0.05, ^##^ *p* < 0.01 compared with the control group; *p < 0.05, **p < 0.01 compared with the CIA group.

To determine the regulatory effect of YSJB on OCs differentiation whether *via* the JAK2/STAT3 pathway, we performed loss-of-function studies in OPCs. OPCs were treated with JAK2 siRNA (#1, #2, and #3) to decrease JAK2 expression. JAK2 mRNA levels were significantly reduced after gene knockdown (Figure 2A). JAK2 siRNA #3 showed the highest knockdown efficiency and was used in subsequent experiments.

The mRNA levels of JAK2 and STAT3 as well as the protein levels of p-JAK2/JAK2 and p-STAT3/STAT3 were measured in single culture systems of OC and Treg respectively (Figures 2B–I), following intervention with YSJB and other reagents. The results showed that the mRNA levels of JAK2 and STAT3 in the CIA group were significantly increased compared with control group in OCs and Tregs. These results suggested that CIA-containing serum promoted the activation of JAK2/STAT3 signaling pathway in OCs and Tregs. However, after treatment with MTX or YSJB, their expression levels were reduced compared with those in the CIA group (Figures 2B,C and Figures 2F,G). Western blot assay showed similar trends in protein expressions of JAK2, STAT3 in OCs and Tregs after treatment with JAK2 siRNA and YSJB (Figures 2D,H). As we all know that, JAK2/STAT3 signaling pathway plays the role mainly by phosphorylation, so the levels of p-JAK2/JAK2 and p-STAT3/STAT3 were used to illustrate the degree of JAK2/STAT3 signal pathway activation. The levels of p-JAK2/JAK2, p-STAT3/STAT3 were significantly higher in the CIA group compared with the control group. In contrast, the relative expression levels of p-JAK2/JAK2, p-STAT3/STAT3 were significantly lower in the MTX and YSJB groups compared with the CIA group. JAK2 siRNA treatments resulted in significantly lower protein levels of p-JAK2, JAK2, p-STAT3, and STAT3 compared with the Mock groups. Furthermore, after treated with the JAK2 siRNA, the relative expression levels of P-JAK2/JAK2 and P-STAT3/STAT3 in CIA group did not increase as much as treated with the Mock, and additional YSJB treatment didn’t exert strong inhibitory effect on p-JAK2/JAK2, p-STAT3/STAT3 as before (Figures 2E–I). These results illustrated that YSJB could block the JAK2/STAT3 signaling pathway in both Tregs and OCs, indicating the role of JAK2/STAT3 pathway in the protection of YSJB regulate bone immune microenvironment.

### YSJB Inhibited Osteoclastogenesis, Perhaps Through JAK2/STAT3 Signaling Pathway

**FIGURE 3:**
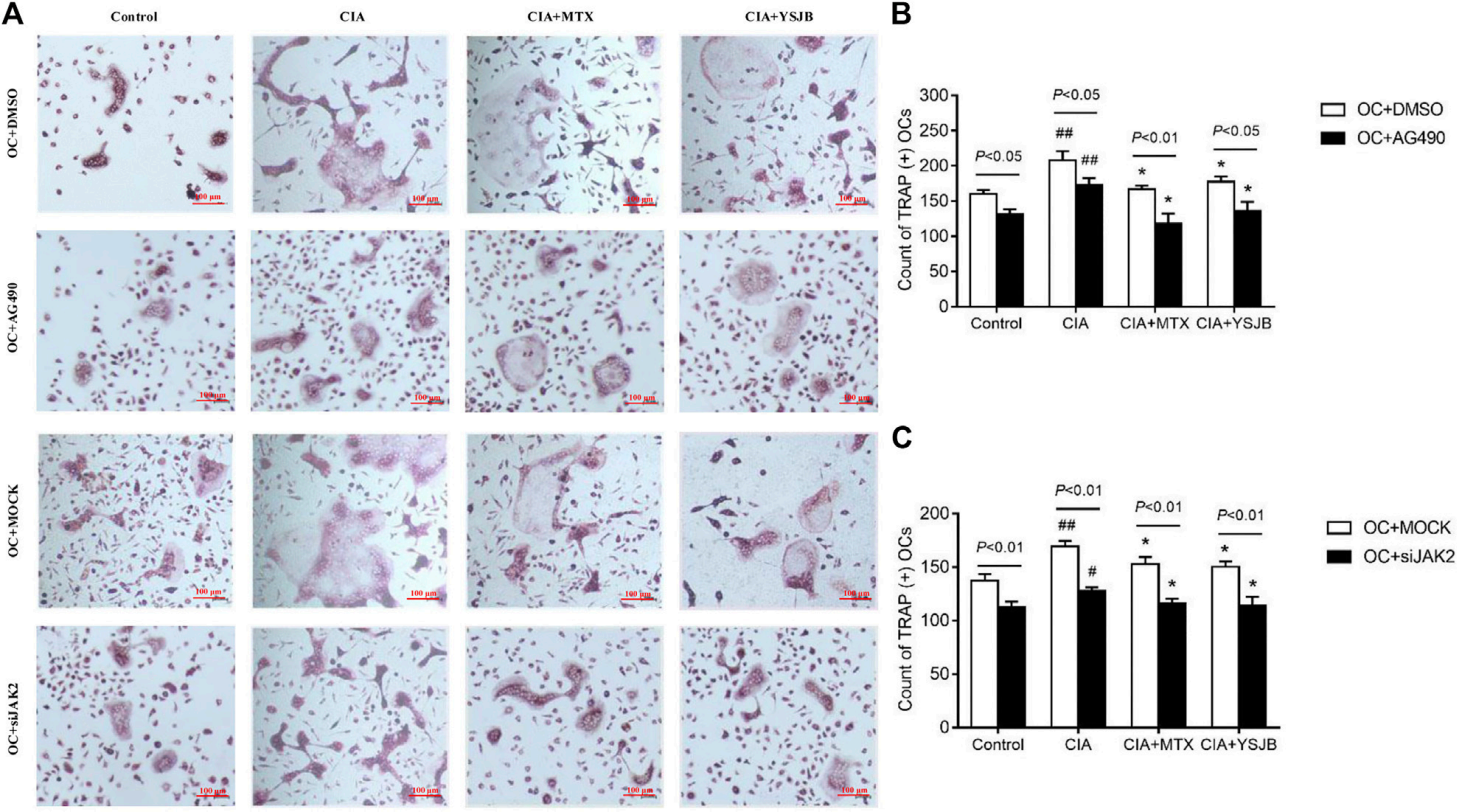
Effect of YSJB, AG490 and JAK2 siRNA on osteoclastogenesis in the OC single culture system. **(A)** Images of TRAP staining in OPCs treated with drug-containing serum (Control, CIA, CIA + MTX, CIA + YSJB), AG490 and JAK2 siRNA. Scale bar = 100 μm. **(B,C)** The number of TRAP^+^ cells. The data are shown as the mean ± SEM (n ≥ 2). ^#^ *p* < 0.05, ^##^ *p* < 0.01 compared with the control group; *p < 0.05; **p < 0.01 compared with the CIA group.

**FIGURE 4:**
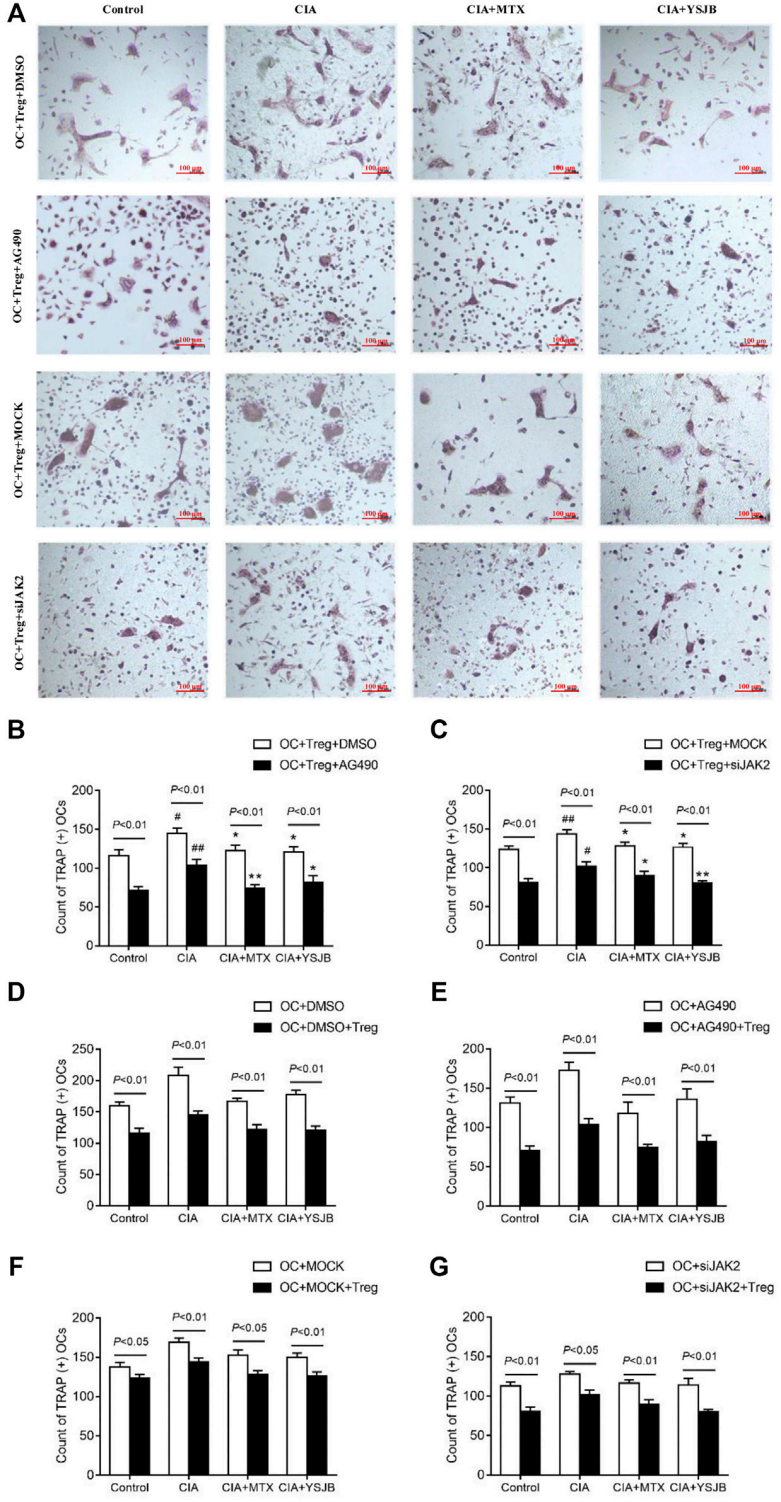
Effect of YSJB, AG490 and JAK2 siRNA on osteoclastogenesis in the OC-Treg coculture system (25:1). **(A)** Images of TRAP staining in the coculture system after treatment with drug-containing serum (Control, CIA, CIA + MTX, CIA + YSJB), AG490 and JAK2 siRNA. Scale bar = 100 μm. **(B,C)** The number of TRAP^+^ cells in the coculture system treated as described in **(A)**. **(D–G)** The number of TRAP^+^ OCs after the addition of Tregs. The data are shown as the mean ± SEM (n ≥ 2). ^#^ *p* < 0.05, ^##^ *p* < 0.01 compared with the control group; *p < 0.05, **p < 0.01 compared with the CIA group.

In order to explore the effect of JAK2/STAT3 signaling pathway on OC differentiation, AG490 or JAK2 siRNA was used in the process of inducing OPCs differentiation into OC. Osteoclastogenesis was evaluated by TRAP staining (Figure 3A and Figure 4A). Interestingly, the number of TRAP^+^ cells was significantly downregulated after treatment with AG490 or JAK2 siRNA in both the OC single culture system and the coculture system. These results suggested that blocking or knocking down JAK2 could inhibit osteoclastogenesis. As shown in Figures 3B,C and Figures 4B,C, the number of TRAP^+^ cells in the CIA group was significantly increased compared with that in the control group in the absence or presence of AG490 and JAK2 siRNA. Compared with that in the CIA group, the number of TRAP^+^ cells in the MTX group and YSJB group decreased. These results suggested that CIA-containing serum promoted osteoclastogenesis, while YSJB and MTX could inhibit the osteoclastogenesis. The combination of YSJB and AG490/JAK2 siRNA did not enhance the inhibitory effect of YSJB on OC differentiation. It might suggest that YSJB could inhibit the differentiation of OCs through CIA-induced JAK pathway. In addition, we found that the number of TRAP^+^ cells was lower in the coculture systems than in the single OC culture system (Figures 4D–G). The results showed that Tregs increased suppression of YSJB in OC formation. This might be related to the fact that YSJB inhibited JAK2/STAT3 signaling pathway in OCs and Tregs.

### YSJB Inhibited OC-Mediated Bone Resorption, Perhaps Through JAK2/STAT3 Signaling Pathway

**FIGURE 5:**
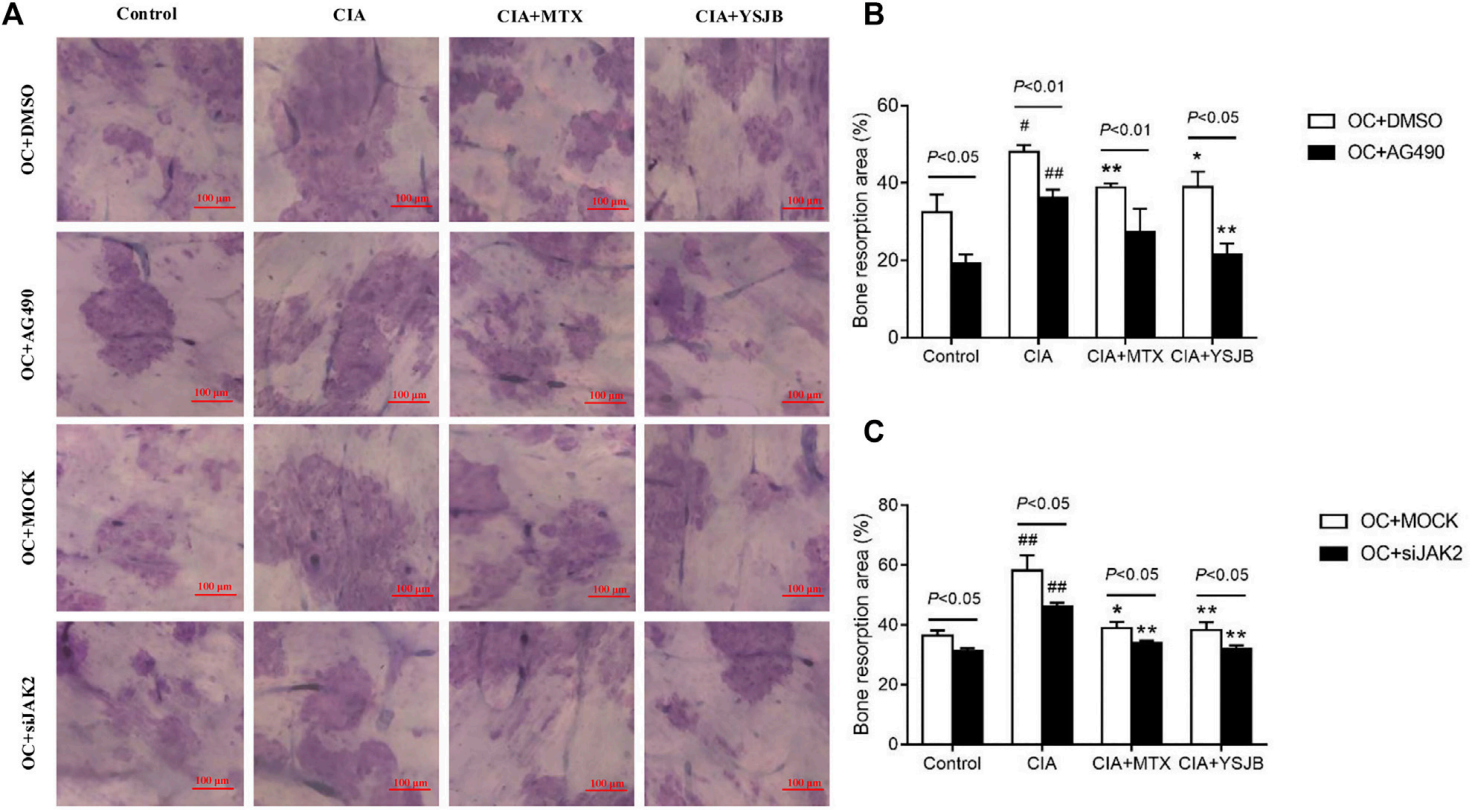
Effect of YSJB, AG490 and JAK2 siRNA on bone resorption in the OC single culture system. **(A)** Images of bone resorption pits. Scale bar = 100 μm. **(B,C)** The areas of the bone resorption pits were measured by the image analysis program. The data are shown as the mean ± SEM (n ≥ 2). ^#^ *p* < 0.05, ^##^ *p* < 0.01 compared with the control group; *p < 0.05, **p < 0.01 compared with the CIA group.

**FIGURE 6:**
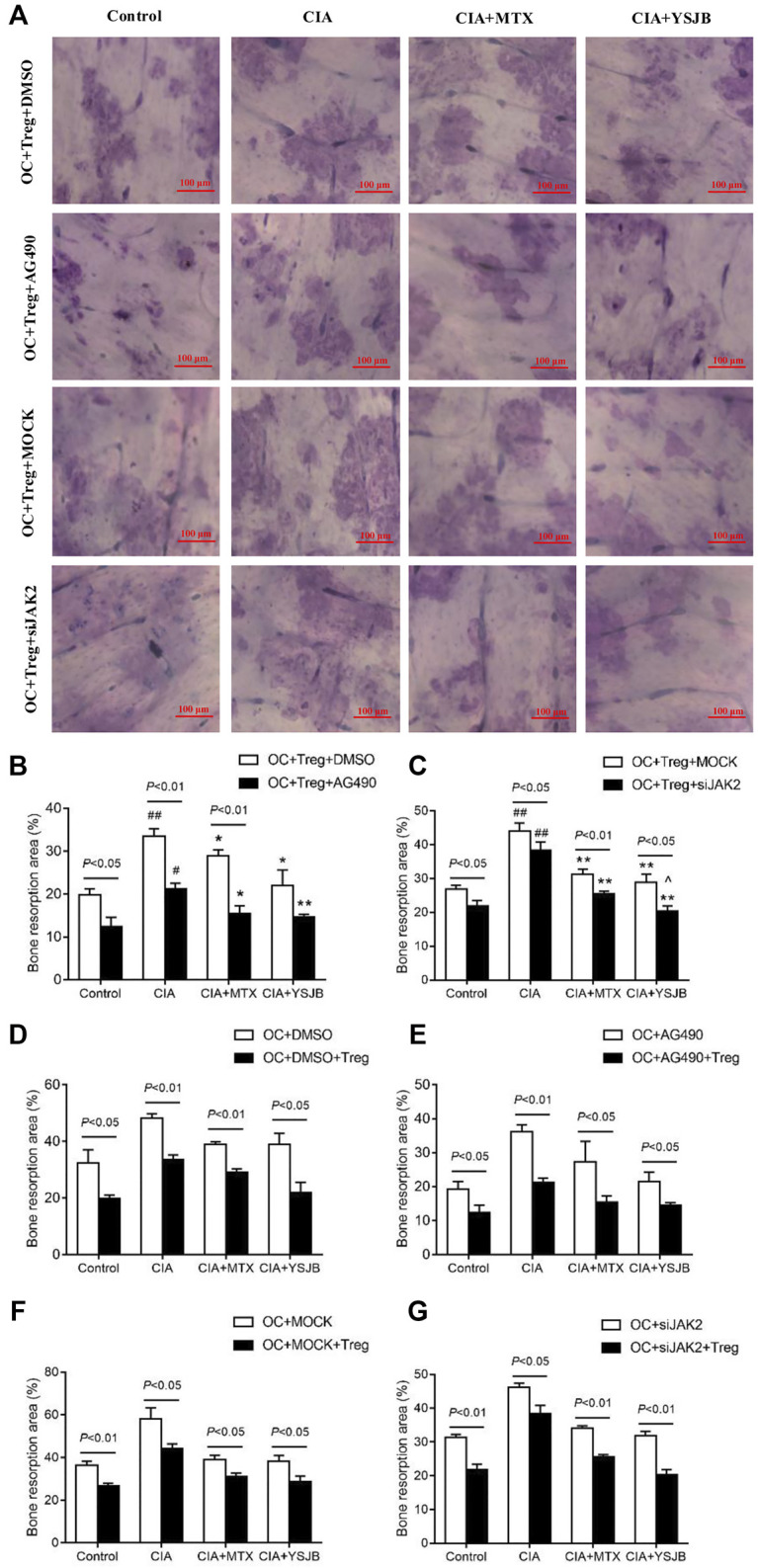
Effect of YSJB, AG490 and JAK2 siRNA on bone resorption in the coculture system. **(A)** Images of bone resorption pits. Scale bar = 100 μm. **(B,C)** The areas of the bone resorption pits were measured by the image analysis program. **(D–G)** The areas of the bone resorption pits after the addition of Tregs. The data are shown as the mean ± SEM (n ≥ 2). ^#^ *p* < 0.05, ^##^ *p* < 0.01 compared with the control group; *p < 0.05, **p < 0.01 compared with the CIA group; ^*p* < 0.05, ^ ^*p* < 0.01 compared with the CIA+MTX group.

After OPCs were cultured for 10 days, the bone slices were stained with toluidine blue. The absorbed surfaces of the bone slices were measured in each group to elucidate the effect of YSJB on bone resorption (Figure 5A and Figure 6A). Consistent with the TRAP staining results, the adsorbed surface was smaller in the presence of AG490 and JAK2 siRNA than in the absence of these factors. It suggested that blocking JAK2/STAT3 signaling pathway could inhibit bone resorption. In addition, in the absence or presence of AG490 and JAK2 siRNA, bone resorption was obviously increased in the CIA group compared with the control group. In the presence of MTX or YSJB, the areas of bone lacunae were significantly reduced. The results illustrated that MTX and YSJB inhibited bone resorption. (Figures 5B,C and Figures 6B,C). Moreover, the presence of Tregs also reduced bone resorption (Figures 6D–G). Perhaps, these due to the inhibitory effect of YSJB on JAK2/STAT3 signaling pathway.

### Blocking the JAK2/STAT3 Signaling Pathway Downregulated RANK, NFATc1, and C-fos mRNA Levels in OCs

**FIGURE 7:**
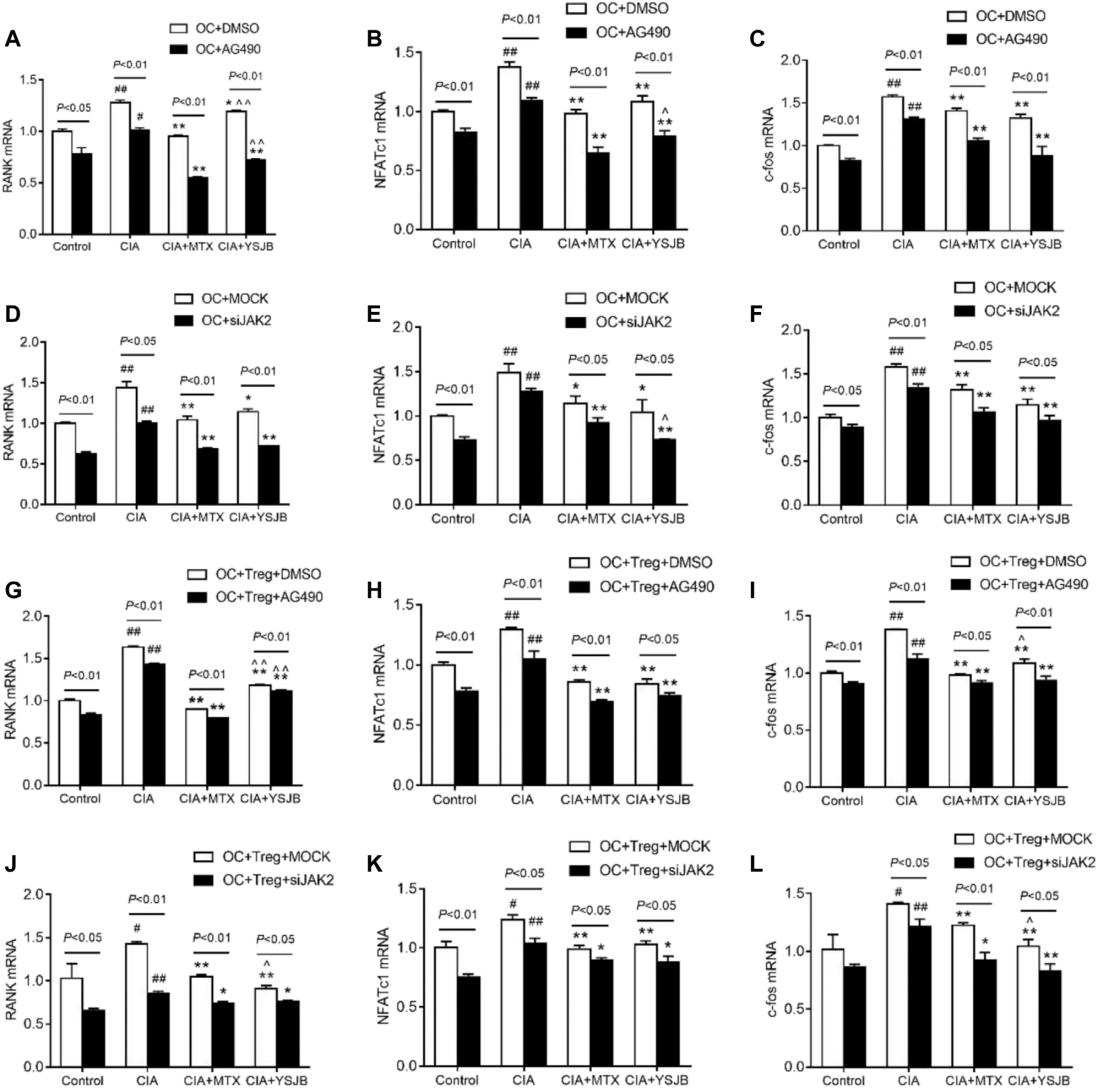
YSJB**,** AG490 and JAK2 siRNA decreased the mRNA levels of RANK, NFATc1, and c-fos, which were associated with the differentiation and maturation of OCs. **(A–F)** The mRNA levels of RANK, NFATc1, and c-fos in the OC single culture system. **(G–L)** The mRNA levels of RANK, NFATc1, and c-fos in the coculture system. Cells were treated with drug-containing serum, AG490 or JAK2 siRNA. ^#^ *p* < 0.05, ^##^ *p* < 0.01 compared with the control group; *p < 0.05, **p < 0.01 compared with the CIA group; ^*p* < 0.05, ^ ^*p* < 0.01 compared with the CIA+MTX group.

As shown previously, OC differentiation and bone resorption were significantly inhibited in the groups with low JAK2 expression. As specific genes associated with OC differentiation, RANK, NFATc1 and c-fos reflect the activity of OCs. To investigate the molecular mechanism by which YSJB, AG490 and JAK2 siRNA regulate the activity of OCs, the mRNA levels of RANK, NFATc1 and c-fos in the OC single culture system and coculture system were measured. We showed that YSJB and MTX significantly suppressed the mRNA expression of RANK, NFATc1 and c-fos both in OC single cultures and in the coculture system compared with those in the CIA group. JAK2 inhibitors and JAK2 knockdown inhibited the expression of these three genes (Figures 7A–L). Collectively, we demonstrated that YSJB restrained the JAK2/STAT3 signaling pathway to inhibit the expression of certain genes associated with OC differentiation.

### Blocking the JAK2/STAT3 Signaling Pathway Promoted Treg Secretion of IL-10

**FIGURE 8:**
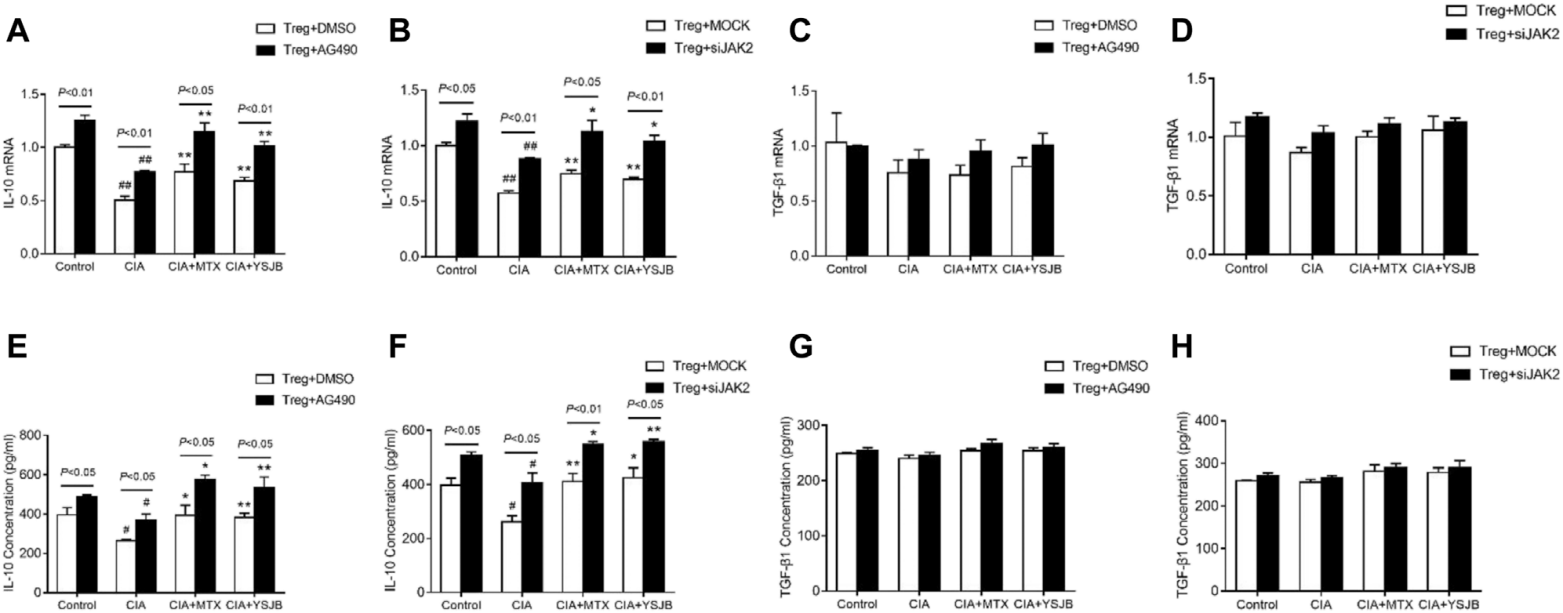
YSJB, AG490 and JAK2 siRNA promoted the secretion of IL-10 by Tregs. The mRNA levels of IL-10 and TGF-β1 were measured by qRT-PCR **(A–D)** and ELISA **(E–H)** in Tregs treated with drug-containing serum, AG490 or JAK2 siRNA. ^#^ *p* < 0.05, ^##^ *p* < 0.01 compared with the control group; *p < 0.05, **p < 0.01 compared with the CIA group.

In previous studies, we demonstrated that IL-10 and TGF-β1 secreted by Tregs inhibited osteoclastogenesis (Xu et al., 2016). In this study, the expression levels of IL-10 and TGF-β1 were measured in a Treg single culture system. As shown in Figures 8A,B, Figurse 8E,F, IL-10 was expressed at low levels in the CIA group compared with the control group. MTX and YSJB reversed this reduction. However, there was no marked difference in TGF-β1 (Figures 8C,D, Figures 8G,H). Consistent with these results, we also showed that AG490 or JAK2 siRNA promoted IL-10 expression (Figures 8A,B, Figures 8E,F). These results indicated that blocking the JAK2/STAT3 signaling pathway upregulated the expression of IL-10. Perhaps the Treg-mediated reduction in the differentiation and bone resorption of OCs is associated with this signaling pathway.

## Discussion

RA is a common autoimmune disease that leads to joint malformation and even loss of joint function. Under normal circumstances, osteoblasts (OBs) and OCs interact to maintain bone homeostasis. However, OCs are overactivated in RA, shifting bone homeostasis toward bone resorption. Therefore, inhibiting OC differentiation in RA patients is of great importance in the treatment of RA. In addition, it has been reported that the immune system interacts with bone tissue (Walsh et al., 2018).

T cells are crucial in regulating the pathogenesis and progression of RA, especially in the initial stage of the autoimmune response and during local inflammatory responses in the joints (Sakaguchi et al., 2003; Komatsu and Takayanagi, 2012; Zerbini et al., 2017). T cells can differentiate into CD8^+^ T cells and CD4^+^ T cells. The latter can differentiate into Th17, Tregs and other subtypes (Srivastava et al., 2018). Both Th17 cells and Tregs are involved in regulating OC differentiation and jointly maintain bone homeostasis. Th17 cells regulate bone mass in two main ways. On the one hand, RANKL is highly expressed in Th17 cells. RANKL binds to RANK on the surface of OPCs to promote OC generation. On the other hand, Th17 cells secrete IL-17, which directly enhances RANKL expression in OBs and synovial fibroblasts, thereby promoting the formation of OCs (Ono and Takayanagi, 2017). The RANKL-RANK signaling pathway is known to be important for OC development. RANKL can be expressed on the surface of stromal cells, OBs and T cells. The binding of RANKL and RANK activates the NF-κB signaling pathway, affecting the function of the immune system and adjusting bone remodeling (Theill et al., 2002; Hanada et al., 2011; Liu and Zhang, 2015). In contrast to Th17 cells, Tregs inhibit the production of OCs by blocking RANKL production. A study showed that the binding of Tregs expressing cytotoxic T lymphocyte-associated antigen-4 (CTLA-4) with CD80/CD86 on the surface of OPCs induces OPC apoptosis (Fischer et al., 2019). Moreover, Tregs can also secrete cytokines with immunosuppressive activity, such as IL-10, which inhibits the differentiation and maturation of OCs by upregulating the secretion of osteoprotegerin (OPG) and downregulating the expression of RANKL (Taylor et al., 2006; Dar et al., 2018). YSJB is a frequently used Chinese medicine for the clinical treatment of RA. Studies have shown that YSJB has a regulatory effect on the immune system. We established a method in which OCs were cultured with Tregs to explore the relationship between bone and the immune system in RA (Xu et al., 2016). In the current study, we used this method to evaluate the efficacy and mechanism of YSJB.

In this study, the number of TRAP^+^ cells and the areas of bone lacunae were elevated in the CIA group. YSJB or MTX significantly diminished these two indicators compared with those in the CIA group. MTX is an anchor drug for RA treatment. Studies have shown that MTX inhibits osteoclastogenesis and inflammation (Ponchel et al., 2014; Kanagawa et al., 2016). Therefore, MTX was used as a positive control drug to explore the therapeutic effect of YSJB. The results showed that MTX and YSJB had similar effects on inhibiting OC differentiation and bone resorption. Moreover, YSJB or MTX inhibited the expression of RANK, NFATc1, and c-fos in OCs and promoted the expression of IL-10 in Tregs. However, YSJB was not as effective in inhibiting RANK as MTX.

Here, we found that the expression of JAK2 and STAT3 was increased in the CIA group, suggesting that the JAK2/STAT3 signaling pathway accelerates osteoclastogenesis. The JAK/STAT pathway, particularly when activated by IL-6/gp130 (Bellido et al., 1997), has been shown to play a role in adjusting osteoclastogenesis (Park et al., 2014; Adam et al., 2020; Sims, 2020). It has been reported that IL-6 can not only stimulate OPCs to transform into OCs but also induce osteoclastogenesis by increasing the expression of RANKL in OBs (Tamura et al., 1993). Currently, some JAK inhibitors have been used to treat RA, such as tofacitinib and baricitinib (Mease et al., 2017; Taylor et al., 2017). Studies have shown that AG490 inhibits the expression of RANK, NFATc1 and TRAP in OCs (Li et al., 2013; Park et al., 2014), which is consistent with our results. In addition, our results suggested that YSJB or MTX could interfere with the expression of JAK2 and STAT3 compared with that in the CIA group, reducing OC differentiation and function. However, in OCs, the expression of STAT3 in the YSJB group showed a downward trend after transfection with JAK2 siRNA, but the effect was not significant, perhaps due to the limited sample size. At the molecular level, blocking the JAK2/STAT3 signaling pathway decreased the expression of critical genes associated with OC formation, including RANK, NFATc1 and c-fos, but increased the secretion of IL-10 by Tregs.

**FIGURE 9:**
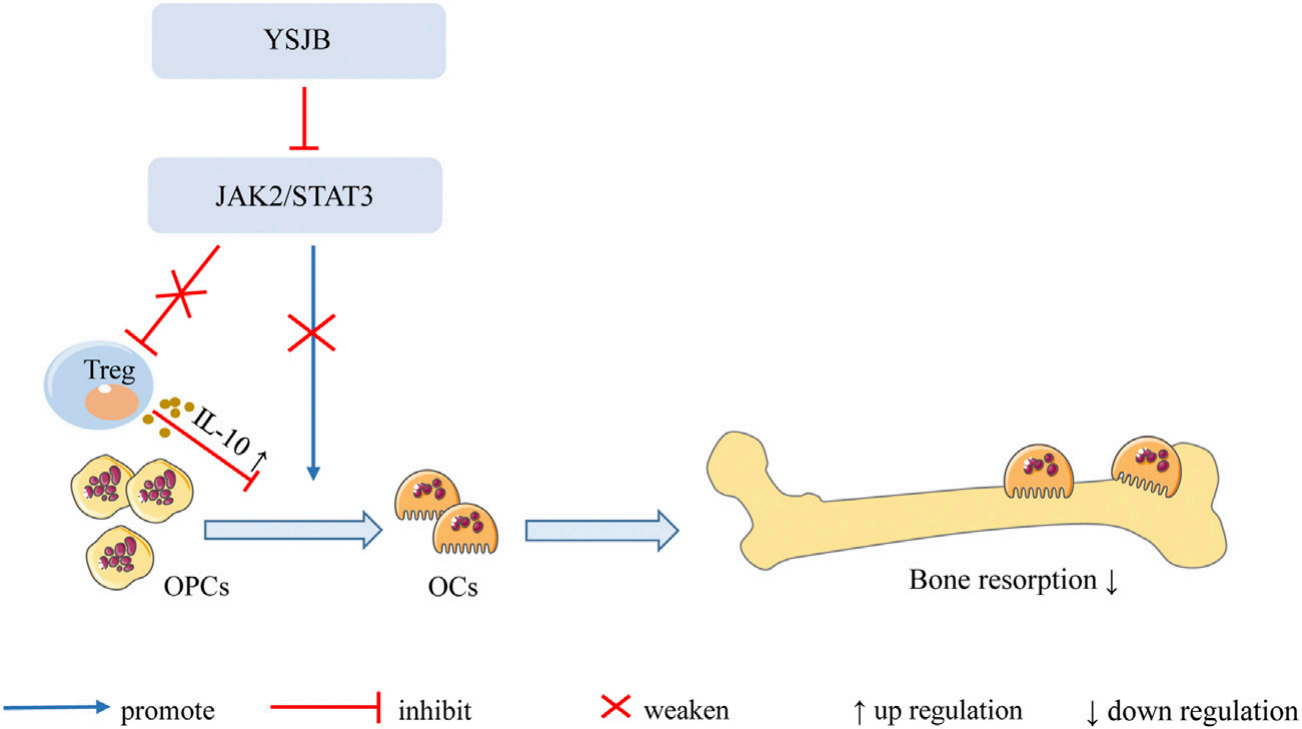
Mechanism of YSJB regulates the bone immune microenvironment. YSJB directly regulates the JAK2/STAT3 signaling pathway in OCs, thereby inhibiting OC differentiation and bone loss. Moreover, YSJB regulates the JAK2/STAT3 signaling pathway in Tregs and upregulates the expression of IL-10. YSJB enhances the immunosuppressive function of Tregs and indirectly inhibits OC differentiation and bone loss.

In summary, our findings demonstrate that YSJB directly regulates the JAK2/STAT3 signaling pathway in OCs and downregulates the expression of RANK, NFATc1 and c-fos in OCs, thereby inhibiting OC differentiation and bone loss. Moreover, YSJB regulates the JAK2/STAT3 signaling pathway in Tregs and upregulates the expression of IL-10. YSJB enhances the immunosuppressive function of Tregs and indirectly inhibits OC differentiation and bone loss (Figure 9).

## Data Availability

The raw data supporting the conclusions of this article will be made available by the authors, without undue reservation.
