# Supplementary material for: Yi Shen Juan Bi Pill Regulates the Bone Immune Microenvironment via the JAK2/STAT3 Signaling Pathway in Vitro
Source: Front Pharmacol. 2021 Dec 14;12:746786. doi: 10.3389/fphar.2021.746786 (PMC8712765; doi:10.3389/fphar.2021.746786)
Supplement: Supplementary file 1 [file DataSheet1.zip › Supplementary Materials/1.DOCX]

**Yi Shen Juan Bi Pill regulates the bone immune microenvironment via the JAK2/STAT3 signaling pathway in vitro**

**Ya Xia, Danping Fan, Xiaoya Li, Xiangchen Lu, Qinbin Ye, Xiaoyu Xi, Qiong Wang, Hongyan Zhao, Cheng Xiao**

**Methods**

The ultra- high performance liquid chromatography (UHPLC) separation was carried out on an UltiMate 3000 liquid chromatograph equipped with a quaternary pump, an online degasser, a thermostatic column compartment (Thermo Scientific, Sunnyvale, CA, USA), and a PAL autosampler (CTC analytics, Zwingen, Switzerland). The Waters ACQUITY BEH C8 analytical column (100 mm × 2.1 mm, 1.7 μm) and the Waters ACQUITY HSS T3 analytical column (100 mm × 2.1 mm, 1.8 μm) were employed, and the column temperature was kept at 50 ℃ during use. An optimized gradient was used at a constant flow rate of 0.35 mL·min−1 using Milli-Q water with 0.1% formic acid (Solvent A) and acetonitrile (Solvent B) in positive ion mode, or using Milli-Q water with 6.5 mM ammonium bicarbonate (Solvent A) and 95% methanol (Solvent B) in negative ion mode. The initial conditions were set at 5% B, and held for 1 min. Then, the gradient was programmed to 100% B at 24 min (in positive ion mode) and kept for 3.5 min or at 18 min (in negative ion mode) and kept for 4 min. Finally, the gradient was returned to the initial conditions at 27.6 min, and then re-equilibrated for 2.5 min to complete the whole run for positive ion mode. For negative ion mode, the gradient was returned to the initial conditions at 22.1 min, and then re-equilibrated for 3 min. A sampling volume of 5 μL was injected for each run with a 200 μL needle wash solvent of 90% aqueous methanol solution. The UHPLC system was used in conjunction with a benchtop Q Exactive hybrid quadrupole-Orbitrap mass spectrometer (Thermo Scientific, Bremen, Germany). In positive ion mode, the ionization of target analytes was enabled via a heated electrospray ionization (HESI) source operated in ESI−mode with the following parameters: capillary temperature of 320 ℃; auxiliary gas heater temperature of 350 ℃; sheath gas, auxiliary gas, and flow rates of 35 and 8 (in arbitrary units); spray voltages of 3.8 kV. Full-scan data within the mass range of mass-to-charge ratio (m/z) 70–1050 was acquired at a mass resolution of 70,000. In negative ion mode, the spray voltage is -3.0 kV.

**Results**

The chemical fingerprint (UHPLC-Q Exactive hybrid quadrupole-orbitrap high resolution accurate mass spectrometry [UHPLC-Q-Orbitrap HRMS]) of YSJB was shown in Supplementary Figure 1 and Supplementary Figure 2.


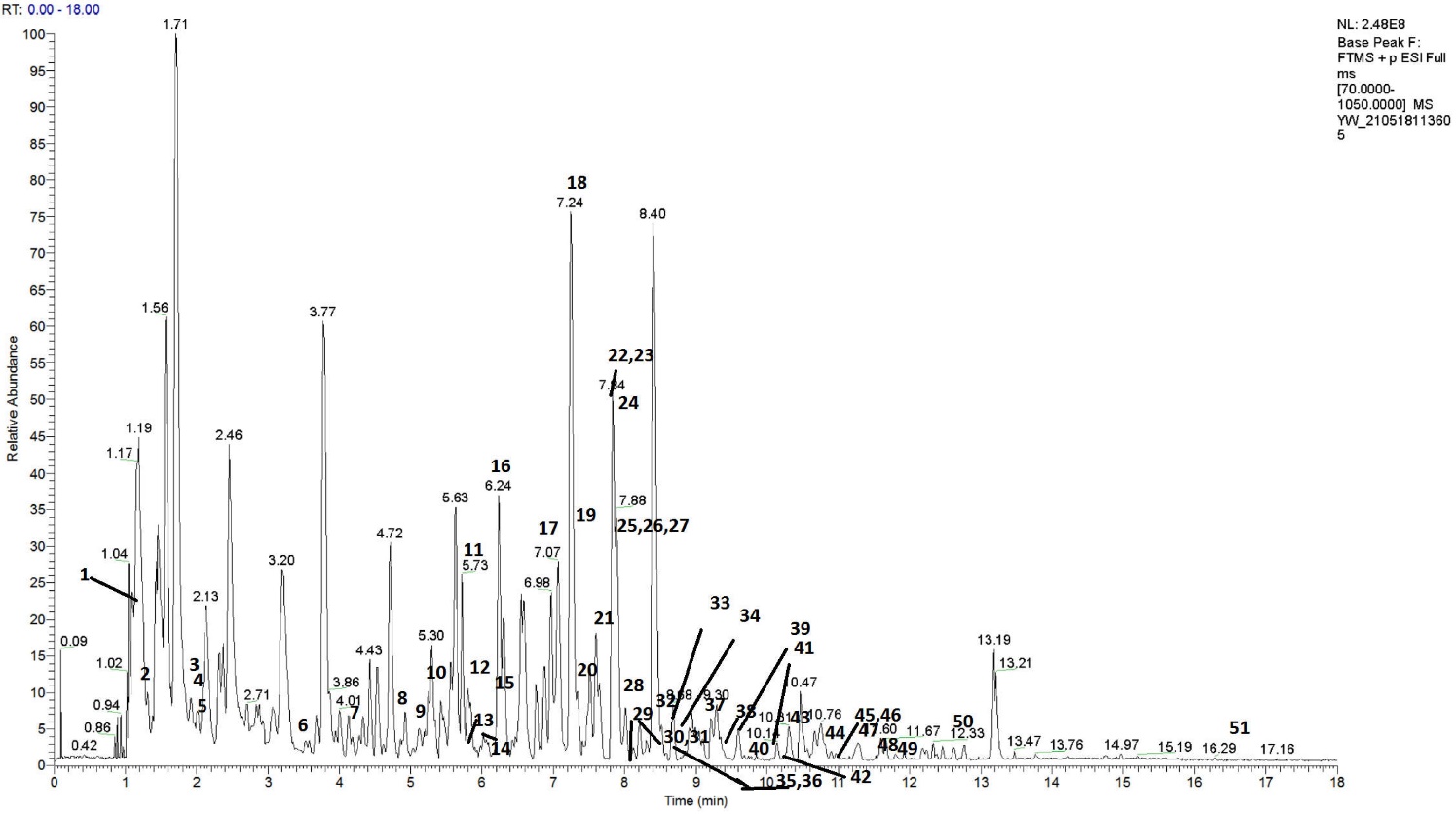
**Supplementary Figure 1** UHPLC-Q-Orbitrap HRMS chemical fingerprint of YSJB (pos-pbc). The specific ingredients are shown in Supplementary Table 1

**Supplementary Table 1** Ingredients of UHPLC-Q-Orbitrap HRMS chemical fingerprint (pos-pbc).

| **Index** | **Herbal Name** | **Chinese Name** | **Ingredient** |
| --- | --- | --- | --- |
| 1 | Scandent hop | Lv Cao | Choline |
| 2 | Ground beeltle | Tu Bie Chong | 2,6-Dimethylnaphthalene |
| 3 | Bombyx batryticatus | Jiang Can | Kynurenine |
| 4 | Angelica sinensis | Dang Gui | Nicotinic acid |
| 5 | Geranium wilfordii | Lao Guan Cao | Biflorin |
| 6 | Angelica sinensis | Dang Gui | trans-Ferulic Acid |
| 7 | Rhizoma drynariae | Gu Sui Bu | Sinapic acid |
| 8 | Ground beeltle | Tu Bie Chong | Naphthalene-1,2-diol |
| 9 | Rhizoma drynariae | Gu Sui Bu | trans-P-Coumaric acid |
| 10 | Rhizoma drynariae | Gu Sui Bu | Isoorientin |
| 11 | Polygonum cuspidatum | Hu Zhang | Resveratrol |
| 12 | Scandent hop | Lv Cao | 6-Hydroxyluteolin |
| 13 | Scandent hop | Lv Cao | Vitexin |
| 14 | Rhizoma Corydalis | Yan Hu Suo | (R)-Canadine |
| 15 | Rhizoma corydalis | Yan Hu Suo | Capaurine |
| 16 | Rhizoma drynariae | Gu Sui Bu | Neoeriocitrin |
| 17 | Rhizoma corydalis | Yan Hu Suo | Stylopine |
| 18 | Polygonum cuspidatum | Hu Zhang | Citreorosein |
| 19 | Angelica sinensis | Dang Gui | Scopoletin |
| **Index** | **Herbal Name** | **Chinese Name** | **Ingredient** |
| 20 | Scandent hop | Lv Cao | Luteone O-glucoside |
| 21 | Caulis spatholobi | Ji Xie Teng | Ononin |
| 22 | Rhizoma corydalis | Yan Hu Suo | Berberine |
| 23 | Epimedium | Yin Yang Huo | Isoliquiritigenin |
| 24 | Rhizoma corydalis | Yan Hu Suo | Corydaline |
| 25 | Rhizoma drynariae | Gu Sui Bu | Eriodictyol |
| 26 | Radix cynanchi panicullati | Xu Chang Qing | Estragole |
| 27 | Rhizoma corydalis, Herba pyrolae | Yan Hu Suo and Lu Xian Cao | Quercetin |
| 28 | Herba pyrolae | Lu Xian Cao | Hydroquinone |
| 29 | Rhizoma drynariae,Herba pyrolae | Gu Sui Bu and Lu Xian Cao | Kaempferol |
| 30 | Rhizoma corydalis | Yan Hu Suo | Physcion |
| 31 | Rhizoma drynariae | Gu Sui Bu | Afzelin |
| 32 | Epimedium, Polygonum cuspidatum | Yin Yang Huo and Hu Zhang | Apigenin |
| 33 | Radix Rehmanniae | Shu Di Huang | dehydro-β-Ionone |
| 34 | Caulis spatholobi | Ji Xie Teng | Genistein |
| 35 | Rhizoma drynariae | Gu Sui Bu | Naringenin |
| 36 | Epimedium | Yin Yang Huo | Icariside I |
| 37 | Epimedium | Yin Yang Huo | Icariin |
| 38 | Ground beeltle | Tu Bie Chong | α-Sinensal |
| 39 | Polygonum cuspidatum | Hu Zhang | Questin |
| 40 | Radix cynanchi panicullati | Xu Chang Qing | Apocynin |
| 41 | Caulis Spatholobi | Ji Xie Teng | Formononetin |
| 42 | Scandent hop | Lv Cao | Apigenin 7,4'-dimethyl ether |
| 43 | Polygonum cuspidatum | Hu Zhang | Coumarin |
| 44 | Radix cynanchi panicullati | Xu Chang Qing | Paeonol |
| 45 | Polygonum cuspidatum | Hu Zhang | Questinol |
| 46 | Caulis spatholobi | Ji Xie Teng | Daidzein |
| 47 | Rhizoma drynariae | Gu Sui Bu | Prunetin |
| 48 | Rhizoma drynariae | Gu Sui Bu | Secoisolariciresinol |
| 49 | Radix cynanchi panicullati | Xu Chang Qing | Damascenone |
| 50 | Epimedium | Yin Yang Huo | Icariside II |
| 51 | Angelica sinensis | Dang Gui | Angelicolide |

#
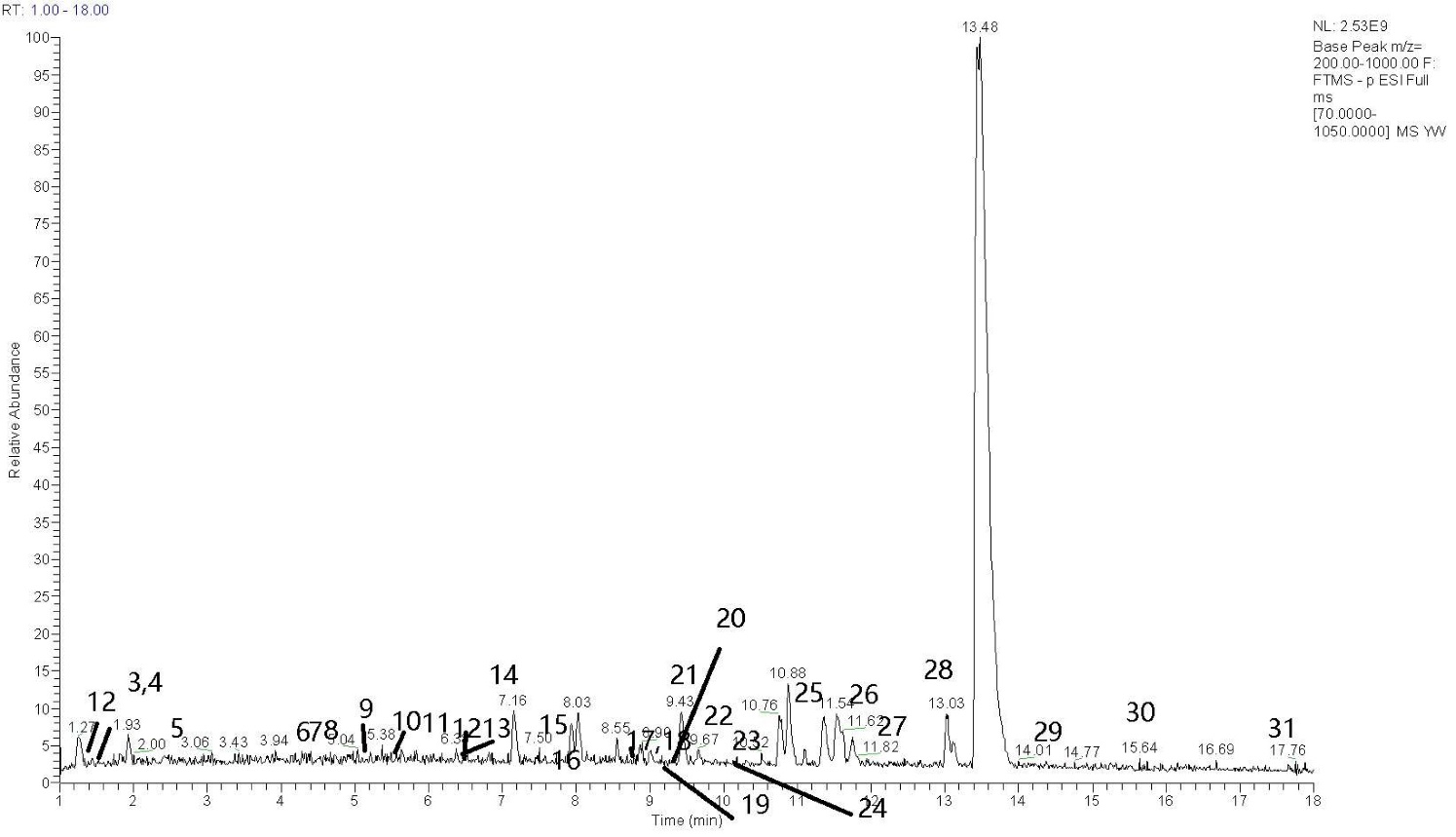
Supplementary Figure 2 UHPLC-Q-Orbitrap HRMS chemical fingerprint of YSJB (neg-pbc). The specific ingredients are shown in Supplementary Table 2.

# Supplementary Table 2 Ingredients of UHPLC-Q-Orbitrap HRMS chemical fingerprint (neg-pbc).

| **Index** | **Herbal Name** | **Chinese Name** | **Ingredient** |
| --- | --- | --- | --- |
| 1 | Radix Rehmanniae | Shu Di Huang | Stachyose |
| 2 | radix rehmanniae recen | Sheng Di Huang | Catalpol |
| 3 | Herba pyrolae | Lu Xian Cao | Hydroquinone |
| 4 | Bombyx batryticatus | Jiang Can | Guanosine |
| 5 | Bombyx batryticatus and Nidus vespae | Jiang Can and Feng Fang | 2',3'-Cyclic AMP |
| 6 | Radix Rehmanniae | Shu Di Huang | Leonuriside A |
| 7 | Nidus vespae | Feng Fang | 2,3-Dihydroxybenzoic Acid |
| 8 | Radix Rehmanniae | Shu Di Huang | Aucubin |
| 9 | Rhizoma drynariae | Gu Sui Bu | Catechin |
| 10 | Epimedium | Yin Yang Huo | Salidroside |
| 11 | Scandent hop | Lv Cao | Vitexin |
| 12 | Geranium wilfordii | Lao Guan Cao | Rutin |
| 13 | Nidus vespae | Feng Fang | 2-Quinolinecarboxylic acid |
| 14 | Rhizoma drynariae | Gu Sui Bu | Eriodictyol |
| 15 | Radix Rehmanniae | Shu Di Huang | Echinacoside |
| 16 | Rhizoma drynariae | Gu Sui Bu | Luteolin 4'-sulfate |
| 17 | Caulis spatholobi | Ji Xie Teng | Daidzein |
| **Index** | **Herbal Name** | **Chinese Name** | **Ingredient** |
| 18 | Rhizoma drynariae and Herba pyrolae | Gu Sui Bu and Lu Xian Cao | Kaempferol |
| 19 | Rhizoma drynariae | Gu Sui Bu | Narirutin |
| 20 | Radix rehmanniae recen | Sheng Di Huang | Acteoside |
| 21 | Polygonum cuspidatum | Hu Zhang | Resveratrol |
| 22 | Angelica sinensis | Dang Gui | Isoeugenitol |
| 23 | Rhizoma drynariae | Gu Sui Bu | Secoisolariciresinol |
| 24 | Rhizoma drynariae | Gu Sui Bu | Orientalone |
| 25 | Ground beeltle | Tu Bie Chong | 5-Amino-6-(1-D-ribitylamino)uracil |
| 26 | Herba pyrolae | Lu Xian Cao | Arbutin |
| 27 | Rhizoma drynariae | Gu Sui Bu | Prunin |
| 28 | Epimedium | Yin Yang Huo | Apigenin |
| 29 | Rhizoma Corydalis | Yan Hu Suo | Physcion |
| 30 | Rhizoma drynariae | Gu Sui Bu | Naringenin |
| 31 | Radix cynanchi panicullati | Xu Chang Qing | Palmitic acid |
