# Supplementary material for: Yi Shen Juan Bi Pill Regulates the Bone Immune Microenvironment via the JAK2/STAT3 Signaling Pathway in Vitro
Source: Front Pharmacol. 2021 Dec 14;12:746786. doi: 10.3389/fphar.2021.746786 (PMC8712765; doi:10.3389/fphar.2021.746786)
Supplement: Supplementary file 1 [file DataSheet1.zip › Supplementary Materials/2.DOCX]

**Yi Shen Juan Bi Pill regulates the bone immune microenvironment via the JAK2/STAT3 signaling pathway in vitro**

**Ya Xia, Danping Fan, Xiaoya Li, Xiangchen Lu, Qinbin Ye, Xiaoyu Xi, Qiong Wang, Hongyan Zhao, Cheng Xiao**

The composition of YSJB is complex. A reliable treatment is the first step in a vitro trial. Therefore, we selected naringin, acteoside, ferulic acid, paeonol, tetrahydropalmatine, monotropein, icariin and polydatin, which are the main components of Rhizoma Drynariae, Radix Rehmanniae Rehmanniae, Angelica Sinensis, Radix Cynanchi Panicullati, Rhizoma Corydalis, Herba Pyrolae, Epimedium and Polygonum Cuspidatum, respectively, as the representative components. The contents and stability of these eight components in YSJB-containing serum were analyzed by high-performance liquid chromatography (HPLC). The detailed methods are illustrated in the following sections.

**1 Materials and methods**

**1.1 Materials**

The reference compounds naringin, acteoside, ferulic acid, paeonol, tetrahydropalmatine, monotropein, icariin and polydatin (structures shown in Supplementary Figure 1) were purchased from Chengdu Chroma-Biotechnology (Chengdu, China) or the National Institutes for Food and Drug Control (Beijing, China). An Agilent 1260 System was used for HPLC. The methanol was HPLC-grade (Aladdin, Shanghai, China).

**1.2 Sample preparation**

Methanol was used to dissolve 1 g of naringin, acteoside, ferulic acid, paeonol, tetrahydropalmatine, monotropein, icariin and polydatin, which were used as reference mixtures with concentrations of approximately 0.2 mg/mL. YSJB powder (0.40 g) was accurately weighed, and an appropriate amount of methanol was added to a 25 mL Erlenmeyer flask. Then, the sample was ultrasonicated for 20 min, followed by the addition of 70% methanol at a constant volume when the sample had cooled to room temperature. Finally, the supernatant was filtered through a 0.45 μm membrane filter. Serum samples were also processed. Two hundred microliters of serum and 600 μL of methanol were placed into a 1.5 mL centrifuge tube and swirled for 10 min to mix thoroughly. Next, the supernatant was separated by centrifugation. The sample was dried with nitrogen and dissolved in methanol again. The supernatant was stored for later analysis.

**1.3 Chromatographic conditions**

An Agilent Poroshell 120 SB-C18 chromatographic column (4.6 mm × 100 mm, 2.7 μm) was used in this study. Pure water (solvent A) and methanol (solvent B) were the mobile phases, and gradient elution (0-25 min, 35%-40% B; 25-40 min, 40%-100% B; 40-50 min, 100% B; 0.4 mL/min) was used. Twenty-microliter samples were injected and analyzed at a column temperature of 25°C and a wavelength of 280 nm.

**2 Results**

**
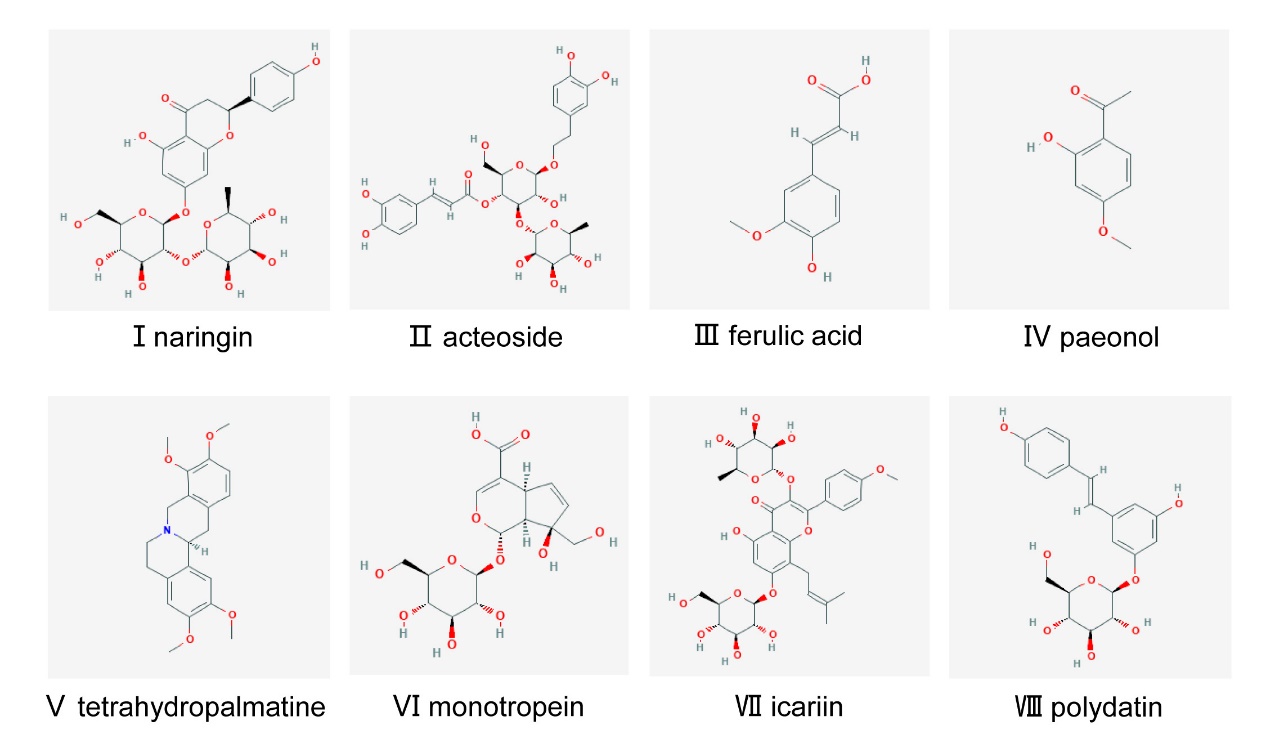
**Five samples of YSJB-containing serum were tested in this experiment. All eight indicators in the reference mixture and YSJB powder solution were successfully separated by HPLC, but not all indicators were detected in YSJB-containing serum (Supplementary Figure 2). Then, the eight compounds in the YSJB powder solution and serum samples were quantitatively measured. A similar level of monotropein was detected in all five serum samples, and a significant level of paeonol was detected in serum sample 1, while no other target compounds were detected in the other serum samples. Moreover, in the YSJB powder solution, the concentration of monotropein was much higher than that of the other target compounds, and the concentration of paeonol was also higher than that of the remaining six compounds, which was consistent with the results of the serum samples (Supplementary Table 1). These results indicated that the YSJB-containing sera were stable and reliable.

**Supplementary Figure 1** Structures of the eight compounds. **Ⅰ:** naringin; **Ⅱ:** acteoside; **Ⅲ:** ferulic acid; **Ⅳ:** paeonol; **Ⅴ:** tetrahydropalmatine; **Ⅵ:** monotropein; **Ⅶ:** icariin; and **Ⅷ:** polydatin.

**
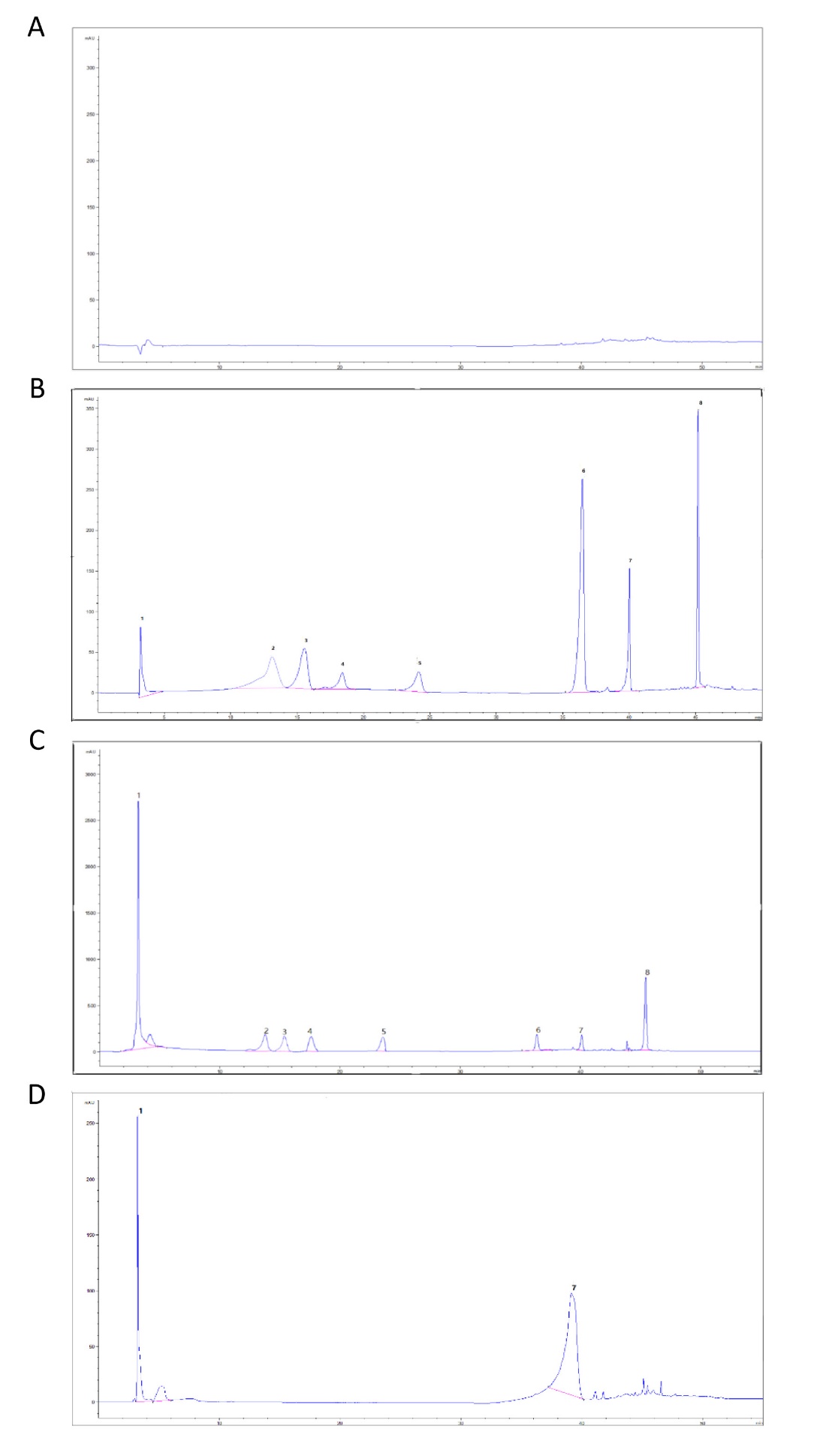
**

**
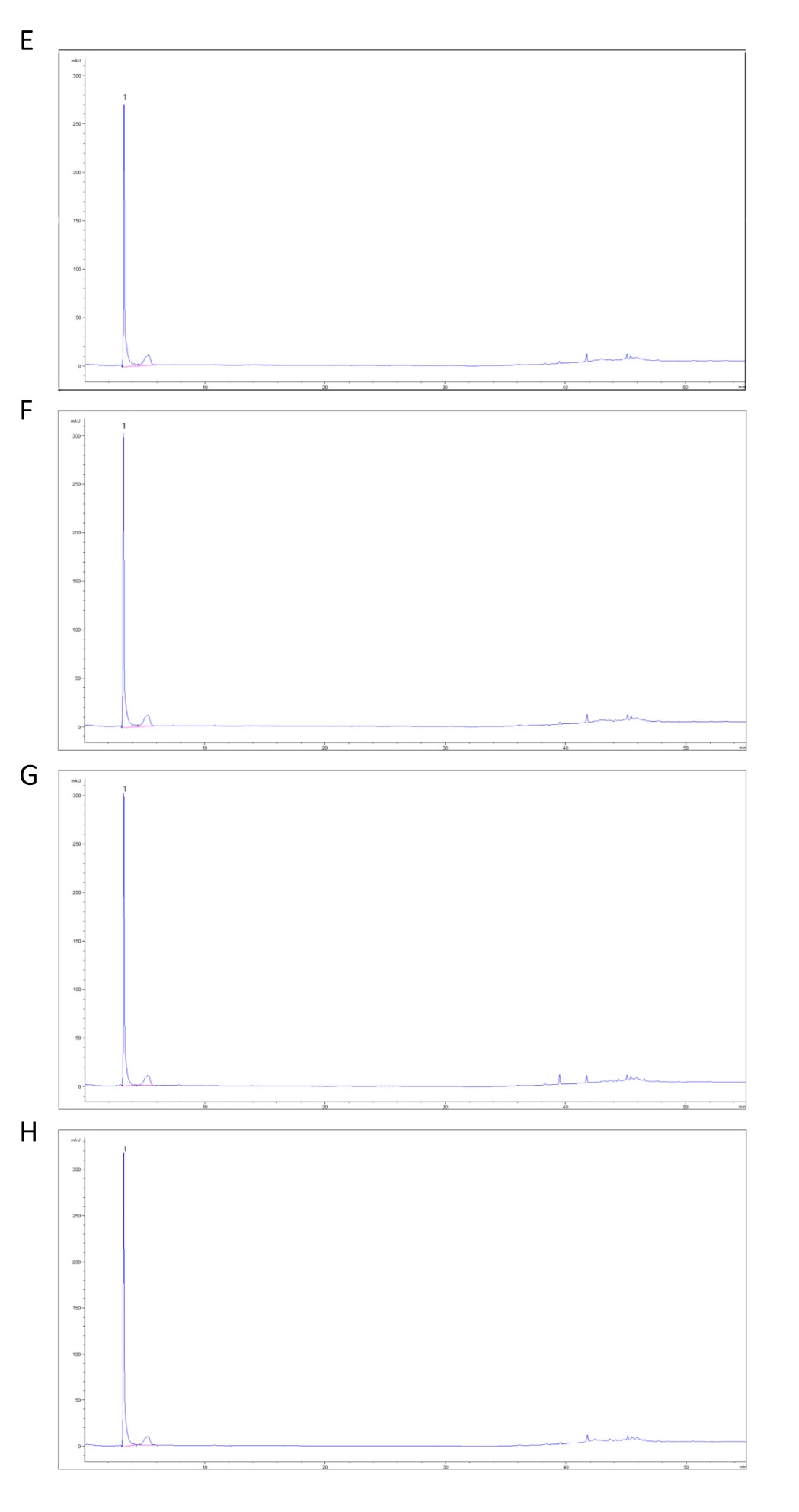
**

**Supplementary Figure 2** HPLC chromatograms. HPLC chromatogram of (A) the blank serum sample; (B) reference mixture; (C) YSJB powder solution; and (D)-(H) serum sample 1, 2, 3, 4, and 5. Peaks：1: monotropein; 2: tetrahydropalmatine; 3: ferulic acid; 4: acteoside; 5: naringin; 6: paeonol; 7: icariin; and 8: polydatin.

**Supplementary Table 1.** Concentration of eight compounds in YSJB powder solution and serum samples (μg/mL).

| Compound | YSJB powder solution | Sample 1 | Sample 2 | Sample 3 | Sample 4 | Sample 5 |
| --- | --- | --- | --- | --- | --- | --- |
| monotropein | 603.4 | 175 | 190 | 191 | 189 | 210 |
| rotundinum | 8.8 |  |  |  |  |  |
| ferulic acid | 2.2 |  |  |  |  |  |
| verproside | 12.1 |  |  |  |  |  |
| naringen | 4.3 |  |  |  |  |  |
| paeonol | 47.5 | 250 |  |  |  |  |
| icariin | 8.6 |  |  |  |  |  |
| emodin | 3.5 |  |  |  |  |  |

Note: Compound concentrations in serum samples in the table = measured concentration * 2
