# Supplementary material for: Yi Shen Juan Bi Pill Regulates the Bone Immune Microenvironment via the JAK2/STAT3 Signaling Pathway in Vitro
Source: Front Pharmacol. 2021 Dec 14;12:746786. doi: 10.3389/fphar.2021.746786 (PMC8712765; doi:10.3389/fphar.2021.746786)
Supplement: Supplementary file 1 [file DataSheet1.zip › Supplementary Materials/3.DOCX]

**Yi Shen Juan Bi Pill regulates the bone immune microenvironment via the JAK2/STAT3 signaling pathway in vitro**

**Ya Xia, Danping Fan, Xiaoya Li, Xiangchen Lu, Qinbin Ye, Xiaoyu Xi, Qiong Wang, Hongyan Zhao, Cheng Xiao**

# Supplementary Table 1. JAK2 siRNA sequences

| PIN | JAK2 siRNA | Sequence (5’ to 3’) | Length |
| --- | --- | --- | --- |
| A0462039 | siJAK2-1 | CUAAGGACUUCAACAAAUATT | 21 |
| A0462040 |  | UAUUUGUUGAAGUCCUUAGTT | 21 |
| A0462041 | siJAK2 -2 | CCUAAGGACUUCAACAAAUTT | 21 |
| A0462042 |  | AUUUGUUGAAGUCCUUAGGTT | 21 |
| A0462043 | siJAK2 -3 | CUUCAGAACAAAUGGUAUUTT | 21 |
| A0462044 |  | AAUACCAUUUGUUCUGAAGTT | 21 |
| A0462047 | FAM negative control | UUCUCCGAACGUGUCACGUTT | 21 |
| A0462048 |  | ACGUGACACGUUCGGAGAATT | 21 |
