# Supplementary material for: Yi Shen Juan Bi Pill Regulates the Bone Immune Microenvironment via the JAK2/STAT3 Signaling Pathway in Vitro
Source: Front Pharmacol. 2021 Dec 14;12:746786. doi: 10.3389/fphar.2021.746786 (PMC8712765; doi:10.3389/fphar.2021.746786)
Supplement: Supplementary file 1 [file DataSheet1.zip › Supplementary Materials/4.DOCX]

**Yi Shen Juan Bi Pill regulates the bone immune microenvironment via the JAK2/STAT3 signaling pathway in vitro**

**Ya Xia, Danping Fan, Xiaoya Li, Xiangchen Lu, Qinbin Ye, Xiaoyu Xi, Qiong Wang, Hongyan Zhao, Cheng Xiao**

# Supplementary Table 1. Primer sequences used for qRT-PCR

| Gene | Primer sequence |
| --- | --- |
| JAK2 | Forward: ACATTCTTACCAAAGTGCGTTC |
|  | Reverse: GCTGAATGAATCTGCGAAATCT |
| STAT3 | Forward: TGTCAGATCACATGGGCTAAAT |
|  | Reverse: GGTCGATGATATTGTCTAGCCA |
| RANK | Forward: CACGGTGGATTCTGAGGGCT |
|  | Reverse: GGGGAGGCAACTGTCACCTT |
| NFATc1 | Forward: TCGGCGGGAAGAAGATGGT |
|  | Reverse: GACTTGGACGGGGCTGGTTA |
| c-fos | Forward: CTTGAAGATGAGAAGTCTGCGTT |
|  | Reverse: CTCTGGGAAGCCAAGGTCAT |
| IL-10 | Forward: GCTCTTACTGACTGGCATGAG |
|  | Reverse: CGCAGCTCTAGGAGCATGTG |
| TGF-β1 | Forward: CTCCCGTGGCTTCTAGTGC |
|  | Reverse: GCCTTAGTTTGGACAGGATCTG |
| β-actin | Forward: AGAGGGAAATCGTGCGTGAC |
|  | Reverse: CCAAGAAGGAAGGCTGGAAAA |
